# Retraction: Preliminary validity of the BNSSS-20 in Arabic: Exploratory study on basic needs satisfaction in sport for a sample of Tunisian athletes

**DOI:** 10.1371/journal.pone.0318591

**Published:** 2025-01-28

**Authors:** The PLOS One Editors

The *PLOS One* Editors retract this article [1] due to concerns about compliance with the PLOS Human Subjects Research policy, the integrity of the underlying data, and the reliability of the published results. The authors’ responses did not resolve these concerns. PLOS regrets that the issues were not identified prior to the article’s publication.

MB did not agree with the retraction. DB responded but expressed neither agreement nor disagreement with the editorial decision. AA, SA, MSK, and ABA either did not respond directly or could not be reached.
